# Exploring Charge-Detection Mass Spectrometry on Chromatographic Time Scales

**DOI:** 10.1021/acs.analchem.3c03325

**Published:** 2023-09-29

**Authors:** Lisa Strasser, Florian Füssl, Tomos E. Morgan, Sara Carillo, Jonathan Bones

**Affiliations:** † Characterisation and Comparability Laboratory, NIBRT − the National Institute for Bioprocessing Research and Training, Foster Avenue, Mount Merrion, Blackrock Co, Dublin A94 X099, Ireland; ‡ MRC Laboratory of Molecular Biology, Francis Crick Avenue, Cambridge Biomedical Campus, Cambridge CB2 0QH, U.K.; § School of Chemical Engineering and Bioprocessing, University College of Dublin, Belfield, Dublin 4, Ireland

## Abstract

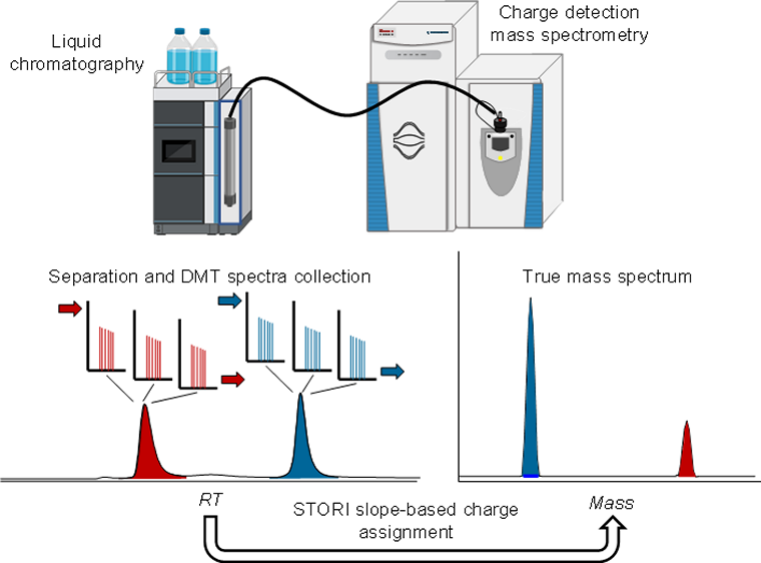

Charge-detection mass spectrometry (CDMS) enables direct measurement of the charge of an ion alongside its mass-to-charge ratio. CDMS offers unique capabilities for the analysis of samples where isotopic resolution or the separation of charge states cannot be achieved, i.e., heterogeneous macromolecules or highly complex mixtures. CDMS is usually performed using static nano-electrospray ionization-based direct infusion with acquisition times in the range of several tens of minutes to hours. Whether CDMS analysis is also attainable on shorter time scales, e.g., comparable to chromatographic peak widths, has not yet been extensively investigated. In this contribution, we probed the compatibility of CDMS with online liquid chromatography interfacing. Size exclusion chromatography was coupled to CDMS for separation and mass determination of a mixture of transferrin and β-galactosidase. Molecular masses obtained were compared to results from mass spectrometry based on ion ensembles. A relationship between the number of CDMS spectra acquired and the achievable mass accuracy was established. Both proteins were found to be confidently identified using CDMS spectra obtained from a single chromatographic run when peak widths in the range of 1.4–2.5 min, translating to 140–180 spectra per protein were achieved. After demonstration of the proof of concept, the approach was tested for the characterization of the highly complex glycoprotein α-1-acid glycoprotein and the Fc-fusion protein etanercept. With chromatographic peak widths of approximately 3 min, translating to ∼200 spectra, both proteins were successfully identified, demonstrating applicability for samples of high inherent molecular complexity.

## Introduction

Classical mass spectrometry (MS) of biomolecules requires either isotopic resolution or the separation of charge states for the determination of the molecular charge (*z*) and mass (*m*) of an ion. Achieving isotopic resolution at high mass-to-charge (*m*/*z*) ratio values on Fourier transform (FT) instruments is challenging due to the inverse relationship between *m*/*z* ratio and resolving power.^1,2^ The separation of charge states becomes difficult with increasing sample heterogeneity as adjacent charge states may overlap, compromising or entirely preventing charge determination. Contrary to conventional MS, charge-detection mass spectrometry (CDMS) is a method that determines the mass of ions directly.^3,4^ In addition to the *m*/*z* ratio, which can be inferred by the frequency of ion oscillations, the charge can be determined through the amplitude of the image current generated by a single ion.^5,6^ Knowledge of both, *m*/*z* ratio and *z* enables the spectral output of molecular weight directly into the mass domain, without the need for subsequent deconvolution procedures. Recently, a charge-detection technique using hundreds to thousands of individual ions detected within an acquisition event, reduced to practice as Direct Mass Technology (DMT) mode, was demonstrated on commercially available Orbitrap instruments and was utilized for the analysis of molecular ions with 6 to 200 positive charges, with charge misassignment rates in the range of 1–4%. Biomolecular structures that have been studied with the aid of CDMS include large proteins, viruses, and other macromolecular entities such as intact ribosomes and nucleic acids.^5−19^ Besides greatly extending the utility range of mass spectrometry, CDMS can produce benefits also seen in individual ion detection MS, significantly enhancing the resolving power of mass spectrometers, as common complications related to ensemble ion FTMS methods, such as ion coalescence and complex beat patterns, can be avoided.^5,12,20,21^

Multiple individual ion detection MS experiments are usually conducted by means of static nano-electrospray ionization (n-ESI)-based direct infusion. Typical acquisition times range from tens of minutes to several hours if high resolving power is desired.^12^ Moreover, these experiments require the delicate tuning of the ion flux into the mass analyzer to allow for the generation of informative mass spectra while avoiding the occurrence of multi-ion events, which impede charge determination. The instrument settings applied are strongly dependent on sample concentration and ionization as well as trapping efficiencies, which complicates automation efforts. This problem was recently addressed through the introduction of automatic ion control (AIC).^22^ This strategy controls the ion flux not based on predefined injection times or a set total maximum number of charges but based on the density of signals along the *m*/*z* axis. Adequate injection times are therefore maintained throughout periods of fluctuating ion magnitudes, which greatly aids the automated analysis of successively introduced samples different in structure, complexity, and abundance. This brings CDMS one step closer to application alongside separation strategies such as liquid chromatography (LC). Such interfacing may be desired in cases where analyte separation, detection with orthogonal detectors, diversion of sample matrices, or enhanced automation capabilities are required.

Here, leveraging AIC, the feasibility of multiple individual ion MS analysis on chromatographic time scales was explored. Direct infusion experiments were performed first to establish a relationship between the number of spectra collectively processed and the molecular masses obtained based on the model protein β-galactosidase (β-gal). Subsequently, a size exclusion chromatography (SEC) method was developed for the separation of β-gal and transferrin, two proteins of moderate heterogeneity with molecular masses of ∼466 and ∼80 kDa, respectively.^12,23^ SEC enables the separation of proteins with fully MS friendly mobile phases of low ionic strength, allowing direct mass spectrometric interfacing.^24^ Moreover, SEC is operated under isocratic conditions, avoiding complications caused by changes of the mobile phase composition throughout the course of a chromatographic run. After initial exploration, the method was then applied for the characterization of a mixture of α-1-acid glycoprotein (AGP) and the Fc-fusion protein etanercept. The heterogeneity of AGP is derived from 5 N-glycosylation sites which can harbor N-glycans of various branching and degree of sialylation, resulting in high protein microheterogeneity.^25^ Etanercept is a homodimer, composed of monomers bearing an IgG1 Fc region fused to a TNF-α receptor, with each monomer containing 3 N-glycosylation and 13 O-glycosylation sites.^26,27^ The combinatorial possibilities of etanercept being equipped with varying numbers and types of glycans create a protein mixture of extreme complexity, higher than can be elucidated by conventional intact LC–MS analysis strategies.

## Experimental

### Materials

Holo-transferrin (human), β-galactosidase (*E. coli*), α-1-acid glycoprotein, GroEL, carbonic anhydrase, and ammonium acetate (99.999%, trace metal basis) were obtained from Sigma-Aldrich (Wicklow, Ireland). The monoclonal antibody nivolumab was obtained from the Hospital Pharmacy Unit of the San Cecilio University Hospital (Granada, Spain). Ultrapure LC–MS grade water was obtained from Fisher (Dublin, Ireland). Etanercept (Enbrel) with a concentration of 50 mg/mL was commercially sourced from Evidentic (Berlin, Germany). Micro Bio-Spin P-6 gel columns in tris buffer were obtained from Bio-Rad (Naas, Ireland).

### Sample Preparation

For static n-ESI direct infusion, β-gal and transferrin were diluted in 100 mM aqueous ammonium acetate to a final concentration of 2 μM. For LC-DMT mode analysis, a sample mixture of transferrin and β-gal in 100 mM ammonium acetate was prepared with concentrations of 0.33 and 0.66 mg/mL, respectively. Etanercept and AGP were prepared as a mixture with concentrations of 0.1 mg/mL each in 100 mM aqueous ammonium acetate. Buffer exchange for samples undergoing direct infusion was performed using Bio-Spin P-6 gel columns according to the manufacturer’s instructions.

### Static n-ESI Direct Infusion

Experiments were performed on a Q Exactive UHMR hybrid quadrupole Orbitrap mass spectrometer (Thermo Fisher Scientific, Bremen, Germany) equipped with an ExD cell (e-MSion, Corvallis, OR, USA), which was autotuned for the transmission of the proteins analyzed. All MS instrument settings applied for native and DMT mode experiments are outlined in Tables S1 and S2.

### LC-DMT Mode Analysis

For protein separation, a Vanquish UHPLC system was used (Thermo Scientific, Germering, Germany). The column employed was an Acquity Protein BEH SEC column with dimensions of 4.6 × 150 mm and a particle size of 1.7 μm (Waters Corporation, Milford, MA, USA). The mobile phase was 50 mM ammonium acetate for transferrin and β-gal and 100 mM ammonium acetate for etanercept and AGP in LC–MS grade water, respectively. Isocratic conditions at a flow rate of 0.05 mL/min were applied. The column temperature was 25 °C and UV detection was performed at 280 nm. The injection amount of the transferrin-β-gal mixture was 3 μL of sample per run corresponding to 1 μg of transferrin and 2 μg of β-gal on column. In case of etanercept and AGP, 0.6 μL were injected, corresponding to 60 ng of protein on column, respectively. The LC system was directly interfaced to the UHMR mass spectrometer through a Thermo Scientific HESI II probe in an Ion Max source. All instrument parameter settings employed are shown in Table S3.

### Native MS Data Analysis

The analysis of native MS data was performed in Thermo Scientific Biopharma Finder 4.1. The model mass range was 10,000–1,000,000, the charge state range was 5–100, and the minimum adjacent charges were 3 to 3. The deconvolution mass tolerance was 20 and 10 ppm, and the target mass was 500,000 and 80,000 Da for β-gal and transferrin, respectively.

### DMT Mode Data Analysis

DMT mode data analysis was performed in STORIboard 1.0 (Proteinaceous, Evanston, Il, USA) using pre-established calibration curves for charge assignment, which were experimentally determined for up to ∼70 charges and extrapolated up to ∼140 charges. Calibration standards included carbonic anhydrase, the monoclonal antibody nivolumab, β-galactosidase, and the protein complex GroEL, which were sprayed in 100 mM ammonium acetate under native conditions. For all proteins analyzed in LC-DMT mode, processing templates were optimized and applied separately. The resolution parameter after processing was adjusted to 0.1× or 0.01×. Data were extracted either based on the most abundant mass peak in each relevant mass region or through application of the fitting mode, allowing for integration of signal clusters. The analysis of successively reduced numbers of spectra presented in Table 1 was enabled through the application of scan range filters. Figure 3B was generated by the collective and simultaneous analysis of ions from of 1–10 runs.

**Table 1:** Relationship between Number of Collectively Processed Spectra Acquired by Static n-ESI-Based DMT Mode Analysis and the Calculated Mass and Mass Deviation Based on Comparison to Results from Native MS Analysis (Δ*m*)

Spectral count	Mass (Da)	Δ*m* (%)
3949	466,940	0.14
3000	467,162	0.19
2000	466,856	0.12
1000	467,066	0.17
500	466,853	0.12
250	467,068	0.17
125	466,740	0.10
50	467,046	0.17

## Results and Discussion

### Static n-ESI Infusion-Based DMT Mode Analysis

The tetrameric protein β-gal was first infused into the Q Exactive UHMR mass spectrometer equipped with DMT mode by means of static n-ESI infusion. Data acquisition was conducted for a total of 32 min at a transient length of 512 ms, corresponding to the acquisition of ∼4,000 DMT mode spectra. Successively reduced numbers of spectra were subsequently utilized for data processing to allow for the establishment of a relationship between spectral count and the true mass obtained. It should be noted that, besides being correctly charge assigned, β-gal was also found to be subject to a series of charge misassignments. These were, however, identified as such and hence not considered for data interpretation. Charge misassignments are further discussed in the Supporting Information (β-galactosidase charge misassignments, Figures S1 and S2).

A table depicting the relationship between the number of collectively processed DMT mode spectra and the determined molecular mass is shown in Table 1. Capabilities for charge and mass assignment did not markedly differ between sets of varying spectral numbers, coherent results were obtained from as few as 50 spectra undergoing processing. At such low spectral numbers, the true mass spectrum appears moderately populated but contains sufficient information for correct mass assignment (Figure S3). The average molecular weight was found to be 466,966 Da, which is in good agreement with data obtained from ensemble ion MS: 466,276 Da with a calculated mass deviation of 0.15%. Within the applied n-ESI setup, 50 spectra required an acquisition time of approximately 0.4 min, suggesting that sufficient data may also be collected during acquisition times corresponding to typical chromatographic peak widths. Details on static n-ESI infusion ensemble ion MS and DMT mode analysis of β-gal can be found in the Experimental section and the Supporting Information.

### LC-DMT Mode Analysis of Transferrin and β-Gal

**Figure 1:**
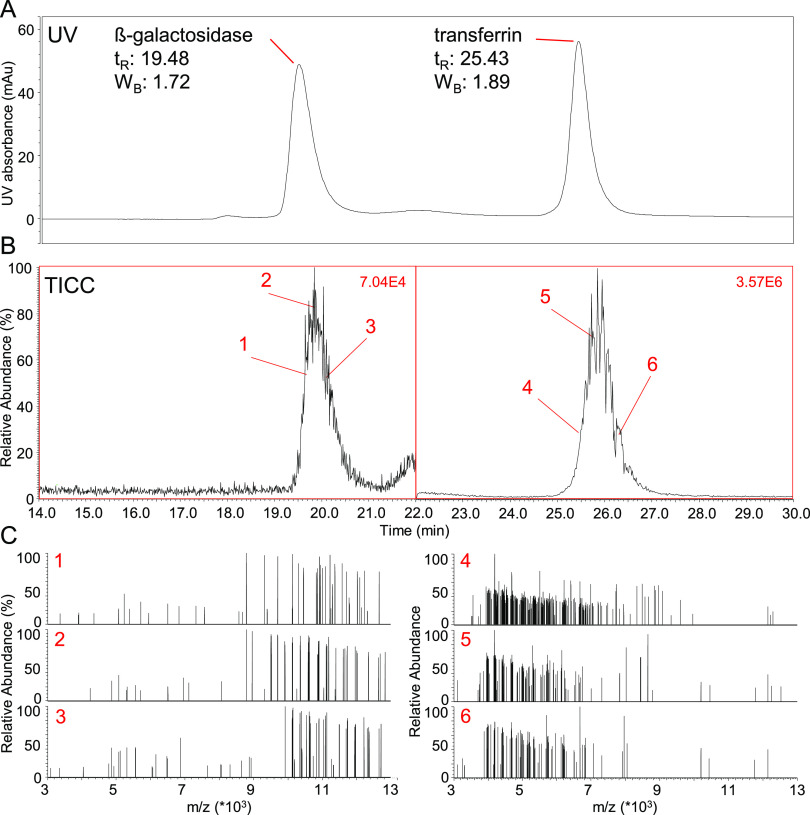
(A) UV chromatogram obtained for the separation of β-gal and transferrin. Metrics such as retention time in minutes (*t*_R_) and peak width at the base in minutes (*W*_B_) are given. (B) TICC corresponding to the UV chromatogram in (A). Both peaks were adjusted to a relative signal intensity of 100%, absolute signal intensities are shown in the right upper corner in red. Numbers from 1 to 6 represent regions where single ion spectra shown in (C) were extracted from. (C) Single ion spectra extracted at retention times indicated in (B). For both proteins, spectra from peak front, apex and tail are shown.

A SEC method was developed and optimized for the separation of β-gal (∼466 kDa) and transferrin (∼80 kDa). Analyte diffusion in SEC can be controlled by adjustment of the internal system volume and the residence time of analytes within the system, which is related to the chromatographic flow rate.^28^ Refinement of both parameters facilitated baseline separation of the two model proteins and resulted in peak widths of 1.4–2.5 min at baseline, respectively (Figure 1A). Liquid chromatography was directly interfaced to DMT mode detection by coupling the LC-UV detector outlet capillary with the ion source of the Q Exactive UHMR mass spectrometer. MS instrument tune settings were optimized for each protein individually and subsequently applied in a time-scheduled manner at relevant elution times. The resolution settings were chosen to maintain acquisition speed at a level that allowed for the acquisition of numerous spectra within the elution time window of each protein, while also attempting to control frequency shifting and charge misassignments. The observed peak widths corresponded to approximately 140–180 spectra for transferrin and β-gal, respectively, which according to results from direct infusion experiments, should be sufficient for correct mass determination and protein identification. The total ion current chromatogram (TICC) corresponding to the UV trace shown in Figure 1A is depicted in Figure 1B. Both peaks are shown with a relative signal intensity adjusted to 100%, although absolute signal intensities varied by a factor of 50, with the transferrin peak dominating the chromatogram. With UV peaks of similar height, the discrepancy between MS peak intensities is likely caused by differences in ionization, ion transmission, and trapping efficiencies for the proteins analyzed. Notwithstanding these differences, the DMT mode spectra of both molecules were well populated with single ion signals, while multi-ion events were kept at a minimum (Figure 1C). Also, highlighting the strength of AIC, spectra of peak fronts and tails were similar in appearance to spectra obtained at the peak apices, as shown in Figure 1C. Single ion spectra of β-gal were populated at a *m*/*z* region of 9,000–13,000 while transferrin ion signals appeared at *m*/*z* 4,000–7,000, reflecting the different molecular masses of both proteins (Figure 1B). Associated averaged spectra taken through peak integration at full width at half maximum are depicted in Figure S4. Details on instrumentation and methods used are provided in the Experimental section as well as in the Supporting Information.

**Figure 2:**
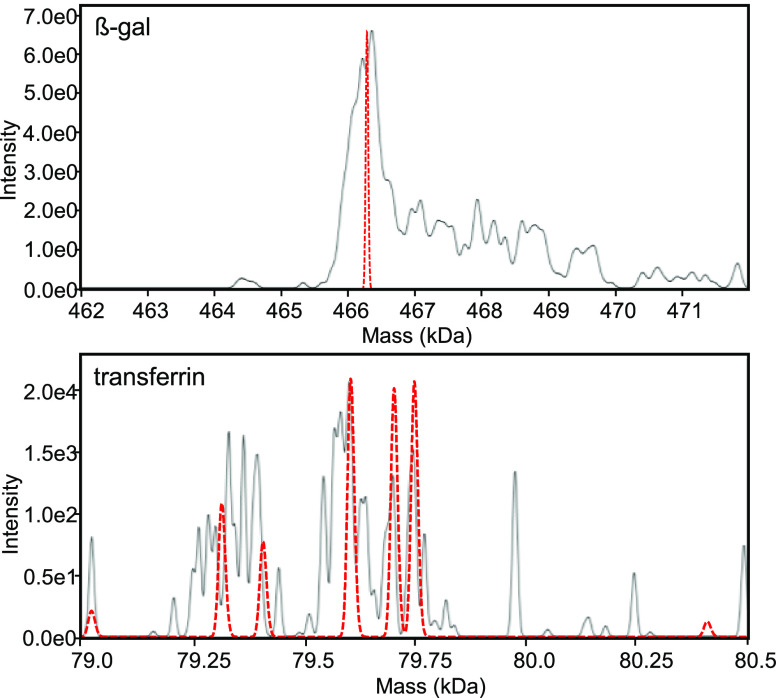
The true mass spectra obtained after the analysis of a single LC-DMT mode run for β-gal (top) and transferrin (bottom) are shown in black. The corresponding deconvoluted spectra obtained through n-ESI-based native MS are shown superimposed in red.

A total of 10 replicate experiments (Figure S5) were performed to evaluate the attainable mass accuracy as a function of spectral count using the LC-DMT mode setup and to assess run-to-run variability. Figure 2 depicts the true mass spectra obtained from a single LC-DMT mode run. The β-gal true mass spectrum (top panel, black trace) showed a peak apex that aligned with the spectra obtained from ensemble ion MS experiments (top, red trace). The difference in peak width may be attributable to differences in desolvation capabilities among both approaches and possible complications during data analysis and processing resulting from frequency shifting and complex beat patters caused by the relatively high density of ion signals. A mitigation strategy could be to reduce the number of ion signals per spectrum, which however, would also entail a decrease in spectral information content which, given the limited time available for acquisition, is counterproductive. The true mass spectrum of transferrin (lower panel, black trace) was dominated by three clusters of peaks which could also be seen in ensemble ion MS spectra (red trace). Peak clusters showed mass offsets of ∼290 Da, indicating differential sialylation.^29^ Peaks within clusters are proposedly derived by differential fucosylation and modification with a previously described 98 Da adduct corresponding to the attachment of sulfuric or phosphoric acid (Figure S6).^23^ The most abundant transferrin peak was found to correspond to an isoform with 19 disulfide bonds, two A2G2S2 type glycans, and a single bound iron atom and showed a mass deviation of 10.0 ppm when compared to the theoretically calculated mass (Figure S6). Upon comparison of native MS and LC-DMT mode data, the abundant peaks in each cluster partially aligned but spectra deviated in terms of the heterogeneity suggested. The sample heterogeneity could be estimated based on the true mass spectrum after LC-DMT mode analysis, but a detailed assignment of protein isoforms was not readily feasible.

**Figure 3:**
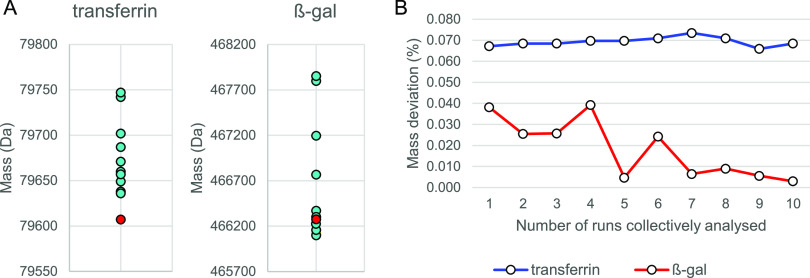
(A) Variation of mass assignment for 10 data files which were individually processed. (B) Mass deviation as a function of the number of cumulatively processed data files. Each data file corresponds to approximately 160 DMT mode spectra per protein.

Data processing of single LC-DMT mode runs resulted in correct assignment of both proteins with mass deviations in the range of 0.036–0.176% for transferrin and 0.006–0.338% for β-gal, based on comparison to masses obtained from native MS analysis. The variation in mass assignment is shown in Figure 3A. Blue data points represent the masses determined by individual processing of 10 LC-DMT mode data files, and the red data points represent masses obtained by native MS. β-gal shows greater overall variation with a relative standard deviation of 0.15% in contrast to 0.05% obtained for transferrin. These results showed that identification of transferrin and β-gal could be attained repeatedly, with deviations of ≤0.18 and 0.34%, respectively. Figure 3B shows mass deviations observed in relation to the number of files cumulatively analyzed. Each file thereby corresponded to approximately 160 DMT mode spectra for either protein and collectively analyzed files to multiples thereof. β-gal showed an inverse relationship between mass deviation and the number of cumulatively processed data files; hence, the mass deviation (%) decreased with an increasing number of spectra. Interestingly, this trend was not observed for transferrin as mass deviations remained steady throughout the experiment.

### LC-DMT Mode Analysis of Etanercept and AGP

**Figure 4:**
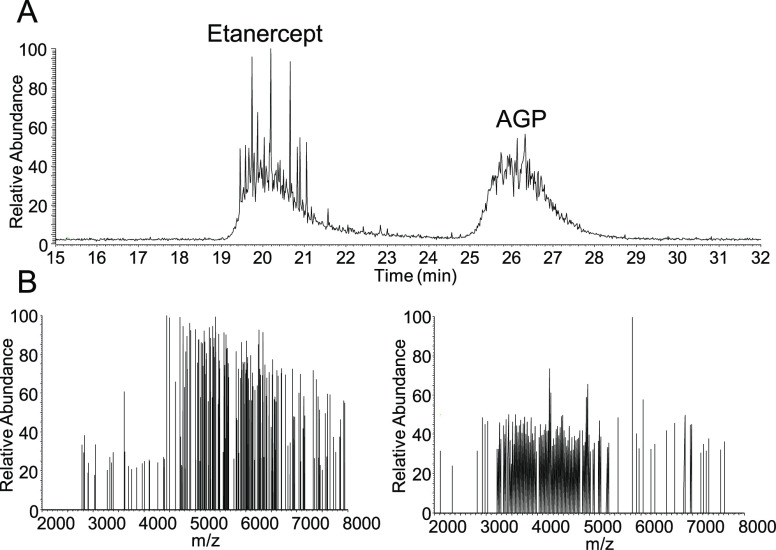
(A) TICC of the SEC separation of etanercept and AGP. (B) Examples of individual DMT mode spectra taken from etanercept (left panel) and AGP peaks (right panel).

While single LC-DMT mode runs were sufficient for mass determination of the two model proteins analyzed, it was unclear if the method is equally applicable to samples typically analyzed in CDMS, i.e., samples of very high complexity. Consequently, a mixture of two complex glycoproteins, namely, AGP and the Fc-fusion protein etanercept, was subjected to LC-DMT mode analysis. Figure 4A depicts the TICC obtained in which both proteins were chromatographically separated. Separation was based on the same SEC setup that was applied for the separation of transferrin and β-gal apart from the use of a mobile phase of higher ionic strength to improve peak shape. Peaks spanned elution times of approximately three minutes in each case, translating to the acquisition of ∼200 DMT mode spectra per protein at a transient length of 1 second. Both proteins featured single ion spectra of good density, with minimal occurrence of multi-ion events as shown in Figure 4B. Single ion signals appeared focused at *m/z* 5,500 and 4,000 for etanercept and AGP, respectively, indicating the different molecular masses of both proteins, which is also in agreement with the chromatographic elution order. Averaged mass spectra after peak integration are shown in Figure S7.

**Figure 5:**
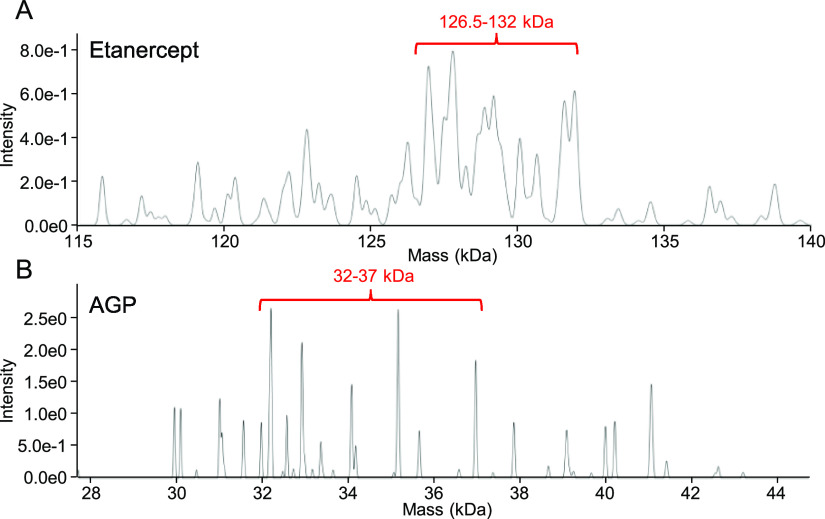
(A) True mass spectrum obtained for etanercept with the mass region featuring the dominant protein signals highlighted in red. (B) True mass spectrum obtained for AGP with the mass region featuring the dominant protein signals highlighted in red.

As was discussed above, both proteins contain several N-glycosylation sites which can be modified by a number of complex glycans of varying monosaccharide composition. Etanercept in addition is modified by multiple O-glycans which represents another layer of complexity, significantly increasing the overall level of protein microheterogeneity. Literature suggests broad mass distributions for either molecule with most abundant forms being of around ∼36 and ∼128 kDa in mass in case of AGP and etanercept, respectively.^27,30^ The dominant mass distributions determined by LC-DMT mode range from 126.5–132.0 and from 32.0–37.0 kDa, respectively, correlating with masses reported in the literature (Figure 5A,B). Predominant masses were found to be 127,637 Da in case of etanercept and 32,213 as well as 35,169 Da for AGP. Assuming theoretical molecular masses of 128 and 36 kDa, the experimentally obtained masses showed deviations of 0.3 and 2.3%, respectively. In terms of the charge distribution, etanercept was found with charge states of between +20 and +26, while AGP featured charge states of +7 to +12 (Figure S8) highlighting the capabilities for the assignment of even low molecular weight proteins with single-digit charge. As was previously shown also for transferrin, the data obtained allowed for an extraction of the predominant masses for both proteins as well as for the approximate assessment of the overall heterogeneity; detailed characterization of individual protein isoforms is, however, not readily feasible on the investigated acquisition time scales.

## Conclusions

Liquid chromatography-charge-detection mass spectrometry enabled mass assignment and therefore the identification of two model proteins, namely, transferrin and β-gal, from a simple mixture. Both proteins were assigned with mass deviations of ≤0.18 and 0.34%, respectively, using data obtained from a single LC-DMT mode run. While the data gathered did not allow for a comprehensive elucidation of the protein microheterogeneity, it was sufficient to estimate the overall protein complexity. It was demonstrated that the mass accuracy obtained can scale with the number of spectra cumulatively analyzed. Based on our observations, this is, however, on the time or spectral count scales investigated, protein specific. The use of LC-DMT mode for the analysis of a mixture of two complex glycoproteins allowed for their identification based on data obtained from a single experiment, suggesting applicability also to other samples of high complexity. In general, it was found that LC-DMT mode acquisition and processing parameters require careful tuning to ensure reliable mass determination which also includes AIC densities. It was however observed that similar densities of between 100 and 200% featured adequate ion injection for all proteins investigated despite differences in molecular mass and concentration. Compromises must be made in terms of ion sampling and transient length. While the sampling of many individual ions at a time allows for the acquisition of highly informative mass spectra, overpopulation can cause a distortion of STORI slopes and complications relating to data processing and mass determination. Long transient times allow for charge determination with good accuracy while also increasing the risk of frequency shifts of ions in the Orbitrap governed by a loss of mass. Such frequency shifts are more likely to occur for large molecules due to their provision of a larger surface area for the association of solvent molecules and counterions, and are further promoted by nonideal desolvation conditions, i.e., at higher infusion flow rates. Sample consumption is clearly lower in case of n-ESI infusion approaches when compared to LC-DMT mode. It was shown however that for complex glycoproteins, high-quality raw data can be obtained with 60 ng of protein on column which can potentially be further reduced if acquisition parameters are optimized for maximum ion sampling. While for some applications, simple direct infusion-based DMT mode experiments may suffice, LC interfacing can offer several advantages. These include the reduction of sample heterogeneity before MS analysis, capabilities to divert MS incompatible sample matrices, possible combination with a series of orthogonal detection and quantitation strategies, superior automation capabilities, and time-staggered sample analysis allowing for the application of more customized MS tune and acquisition parameters. Highly complex samples which fail to be characterized by standard MS strategies may be analyzed using DMT mode if the acquisition of high-quality spectra can be ensured. LC and other means of separation, if further developed, may become standard applications for use with DMT mode. This study forms a fundamental proof of concept for future hyphenation attempts.

## Supporting Information Available

Experimental details on native MS, DMT mode and LC-DMT mode runs; discussion of β-gal charge misassignments; true mass spectrum of β-gal based on 50 DMT mode spectra; averaged LC-DMT mode spectra of transferrin and β-gal; similarity assessment of transferrin and β-gal LC-DMT mode runs; native MS-based annotation of transferrin; LC-DMT mode analysis of etanercept and AGP (PDF)
