# Supplementary material for: Exploring Charge-Detection Mass Spectrometry on Chromatographic Time Scales
Source: Anal Chem. 2023 Sep 29;95(40):15118–24. doi: 10.1021/acs.analchem.3c03325 (PMC10568534; doi:10.1021/acs.analchem.3c03325)
Supplement: Supplementary file 1 — ac3c03325_si_001.pdf [file ac3c03325_si_001.pdf]

Supporting Information

Exploring Charge-Detection Mass Spectrometry on Chromatographic Time Scales

Lisa Strasser,[†,#] Florian Füssl,[†,#] Tomos E. Morgan,[†,‡] Sara Carillo[†] and Jonathan Bones[†,§]

[†]Characterisation and Comparability Laboratory, NIBRT – the National Institute for Bioprocessing Research and Training, Foster Avenue, Mount Merrion, Blackrock, Co. Dublin, A94 X099, Ireland.

[‡]MRC Laboratory of Molecular Biology, Francis Crick Avenue, Cambridge Biomedical Campus, Cambridge CB2 0QH, UK

[§]School of Chemical Engineering and Bioprocessing, University College of Dublin, Belfield, Dublin 4, Ireland.

[#]These authors contributed equally.

Contents

Experimental S3
ß-galactosidase charge misassignments S4
True mass spectrum of β-gal based on 50 DMT mode spectra S5
Averaged LC-DMT mode spectra of transferrin & β-gal S5
Similarity assessment of transferrin & β-gal LC-DMT mode runs S6
Native MS-based annotation of transferrin S7
LC-DMT mode analysis of etanercept and AGP S8

Experimental

Table S1: Instrument parameter settings used for static nano-ESI based native MS of β-galactosidase and transferrin.

**Parameter**	**ß-galactosidase**	**Transferrin**
Spray voltage	0.9 kV	1.0 kV
Capillary temperature	250˚C	250˚C
S-lens RF level	200	200
Resolution setting at m/z 400	6,250	12,500
Trapping gas	Nitrogen	Nitrogen
UHV pressure (mbar)	5.2e-10	1.7e-10
IST desolvation voltage	-200 V	-150 V
Acquisition time	2 min	2 min
Averaging	on	on

Table S2: Instrument parameter settings used for static nano-ESI based DMT mode analysis of β-galactosidase.

**Parameter**	**ß-galactosidase**
Spray voltage	0.9 kV
Capillary temperature	250˚C
S-lens RF level	200
Resolution setting at m/z 400	50,000
Trapping gas	Nitrogen
UHV pressure (mbar)	2.6e-11
IST desolvation voltage	-200 V
Acquisition time	32 min
Averaging	off
Detector m/z optimization	High
Ion transfer target	High
Ion Injection	IT (50 ms)

Table S3: Instrument parameter settings used for LC-DMT mode analysis of β-galactosidase, transferrin, etanercept and AGP.

**Parameter**	**ß-galactosidase**	**Transferrin**	**Etanercept**	**AGP**
Spray voltage	3.8 kV	3.8 kV	3.8 kV	3.8 kV
Capillary temperature	300˚C	300˚C	300˚C	300˚C
Probe heater temp.	150 ˚C	150 ˚C	150 ˚C	150 ˚C
S-lens RF level	200	200	200	200
IST desolvation	-150 V	-100 V	-200 V	-200 V
Ion Flux Control	AIC (200%)	AIC (100%)	AIC (100%)	AIC (100%)
Resolution at m/z 400	50,000	100,000	100,000	100,000
Trapping gas	Nitrogen	Nitrogen	Nitrogen	Nitrogen
Trapping gas pressure (mbar)	5.2e-10	5.0e-11	6.7e-11	6.7e-11
Detector m/z optimization	High	High	High	High
Ion transfer target	High	High	High	High

ß-galactosidase charge misassignments

ß-galactosidase spectra were assigned to several different charge ranges resulting in a true mass spectrum as is depicted in Figure S1. The most intense peak corresponds to the protein with the expected molecular mass of ~466 kDa. Halo peaks appear as repeating patterns suggesting that signals are caused by charge misassignments. Charge heatmaps of the three dominant peaks are shown in Figure S2. It becomes apparent that signals are identical but are shifted by +/-1 charges, respectively. The +/-1 charge assignment will cause a misassignment of the mass by +/- of the detected *m/z* ratio. In consequence, halo peaks are spaced by 9-10 kDa.

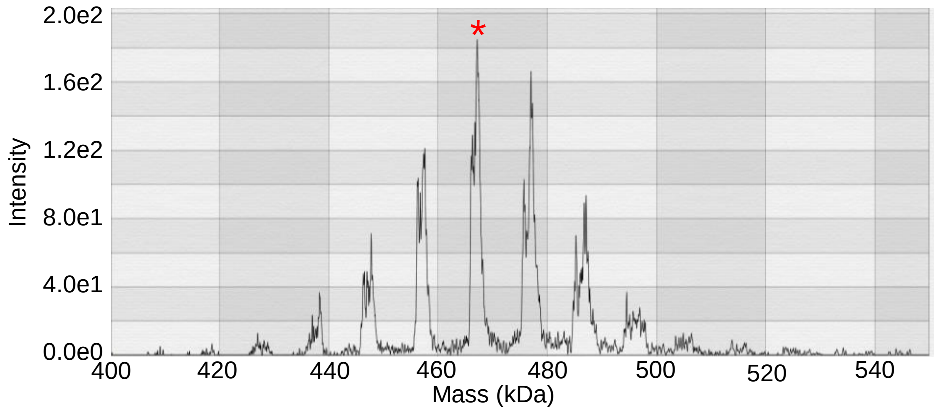

Figure S1: Repeating pattern obtained for the direct mass spectrum of ß-galactosidase. The peak at ~466 kDa (labelled in red) represents the expected molecular mass.

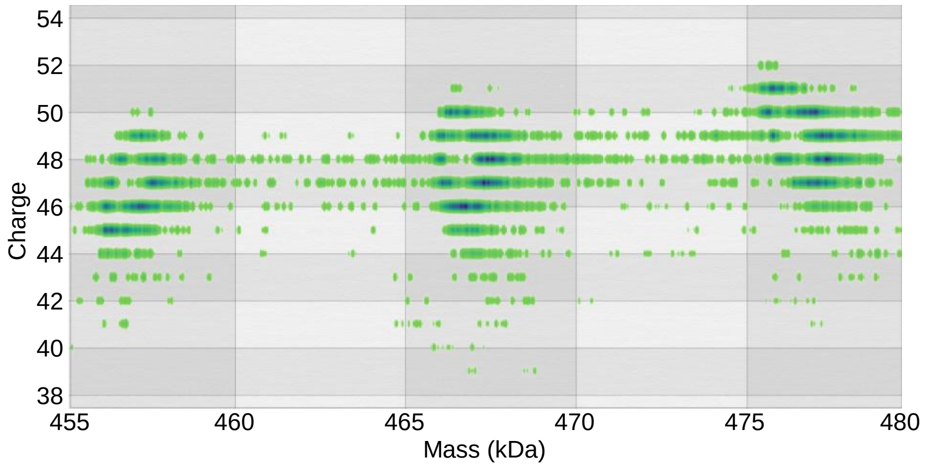

Figure S2: Charge heat map of the three most abundant peaks from Figure S1. The pattern seen is the same across all three peaks with only the charge shifting by one, indicating that the same signals were assigned to multiple charges.

True mass spectrum of β-gal based on 50 DMT mode spectra

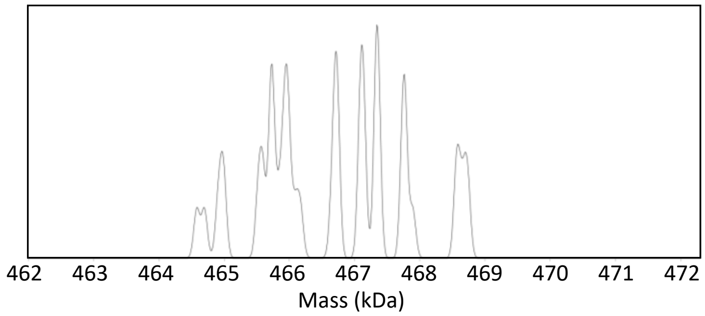

Figure S3: True mass spectrum obtained after processing of 50 DMT mode spectra of β-gal.

Averaged LC-DMT mode spectra of transferrin & β-gal

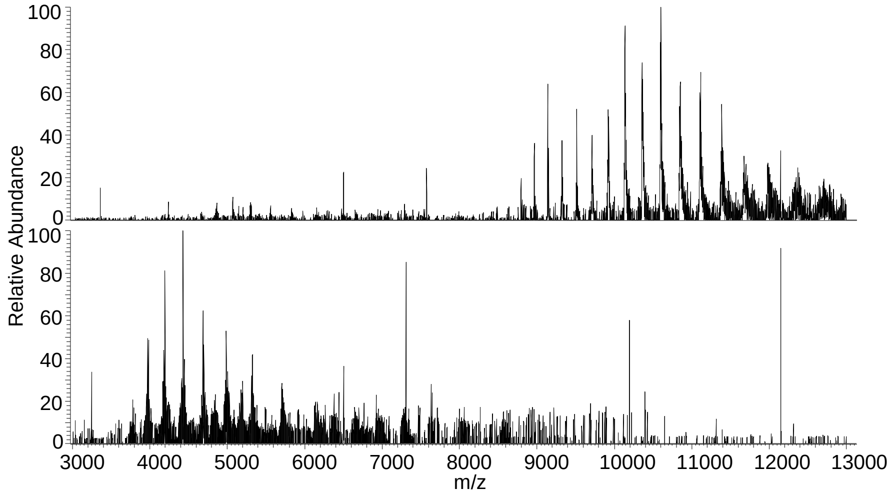

Figure S4: Averaged MS spectra of ß-galactosidase (top) and transferrin (bottom) after full width at half maximum peak integration.

Similarity assessment of transferrin & β-gal LC-DMT mode runs

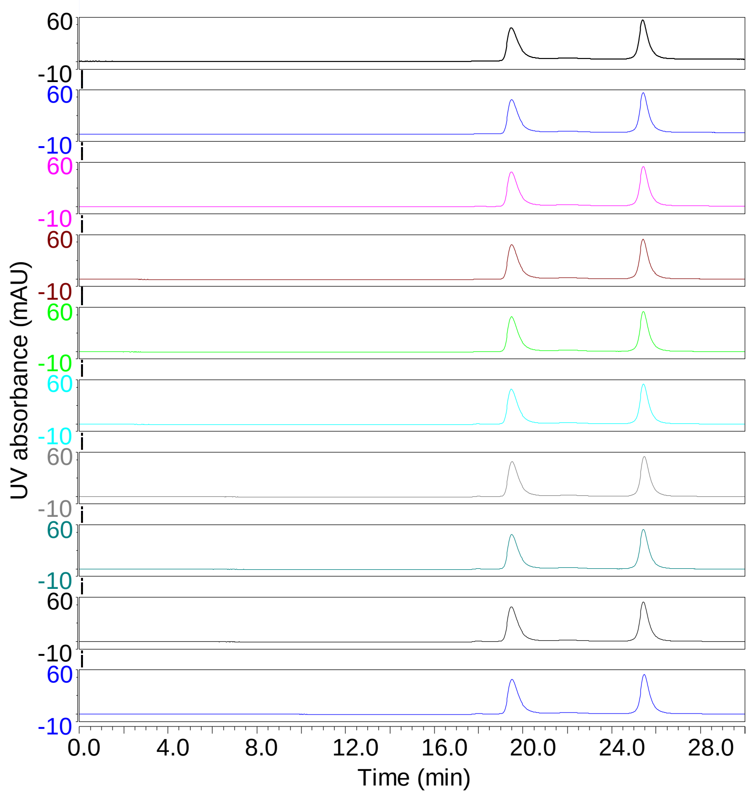

Figure S5: Full chromatographic time scale for ten replicate runs of a ß-galactosidase and transferrin mixture.

Native MS-based annotation of transferrin

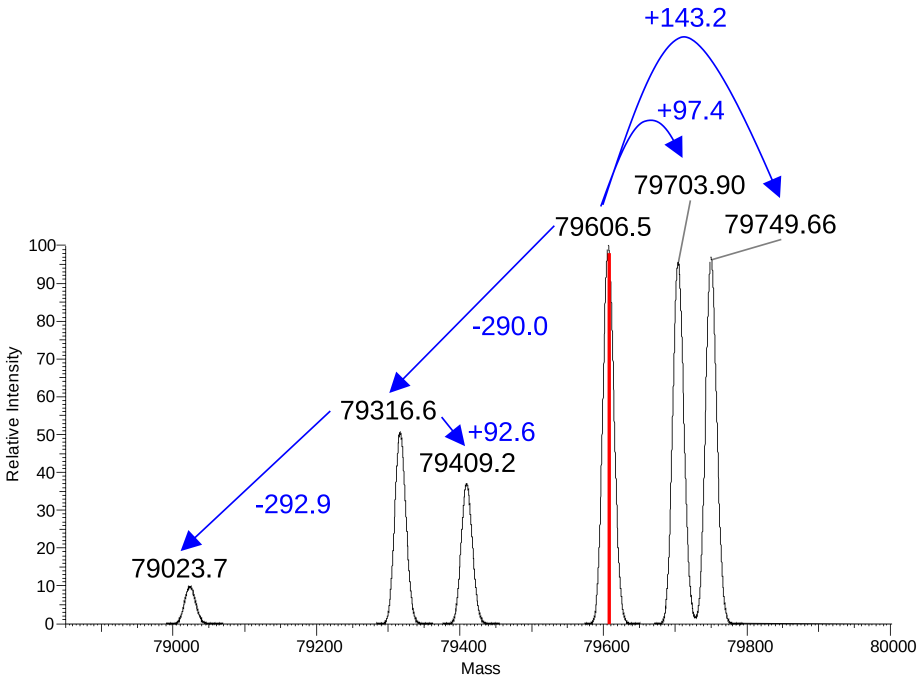

Figure S6: Deconvoluted spectrum of transferrin. Mass shifts in relation to the main peak are outlined in blue. Mass shifts are proposedly corresponding to desialylation (-291 Da), fucosylation (+146 Da) and sulphuric or phosphoric acid adduction (+98 Da). The theoretical mass calculated for the most abundant transferrin peak (79,606.5 Da) is shown in red and amounts to 79,607.3 Da. The calculation was based on the primary sequence mass, 19 disulphide bonds, two N-glycans of the A2G2S2 type and a single bound Fe^{3+}.

LC-DMT mode analysis of etanercept and AGP

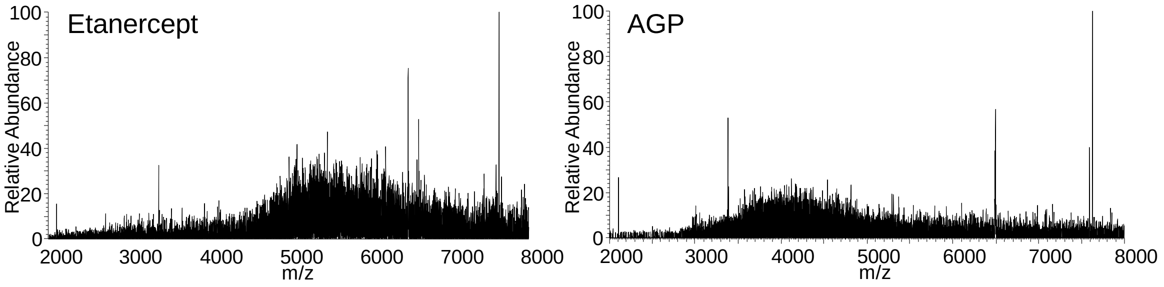

Figure S7: Averaged mass spectra obtained for both proteins after chromatographic peak integration.

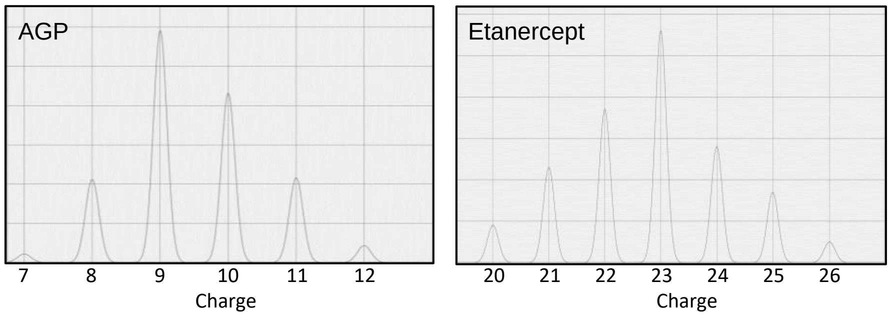

Figure S8: Charge state distribution of AGP and etanercept.
